# Isotope effects in mechanistic studies of l-tyrosine halogen derivatives hydroxylation catalyzed by tyrosinase

**DOI:** 10.1007/s10967-017-5526-1

**Published:** 2017-10-23

**Authors:** Małgorzata Pająk, Marianna Kańska

**Affiliations:** 1 Department of Chemistry, Warsaw University, Pasteur 1 Str., 02-093 Warsaw, Poland; 2 Department of Biochemistry, 2nd Faculty of Medicine, Medical University of Warsaw, 61 Zwirki i Wigury Av., 02-091 Warsaw, Poland

**Keywords:** Deuterium, Halogenated derivatives of l-tyrosine, Isotope effects, Tyrosinase

## Abstract

The kinetic (KIE) and solvent (SIE) isotope effect methods were used to investigate the mechanism of enzymatic hydroxylation of halogenated derivatives of l-tyrosine to l-DOPA catalyzed by the enzyme tyrosinase (EC 1.14.18.1). The values of deuterium KIE and SIE were obtained using the non-competitive method with spectrophotometric measurements. The Lineweaver–Burk plots were used for determination of the inhibition mode of 3′-iodo-l-tyrosine. Based upon kinetic effects values the mechanism of action of enzyme tyrosinase was proposed.

## Introduction

**Fig. 1:**
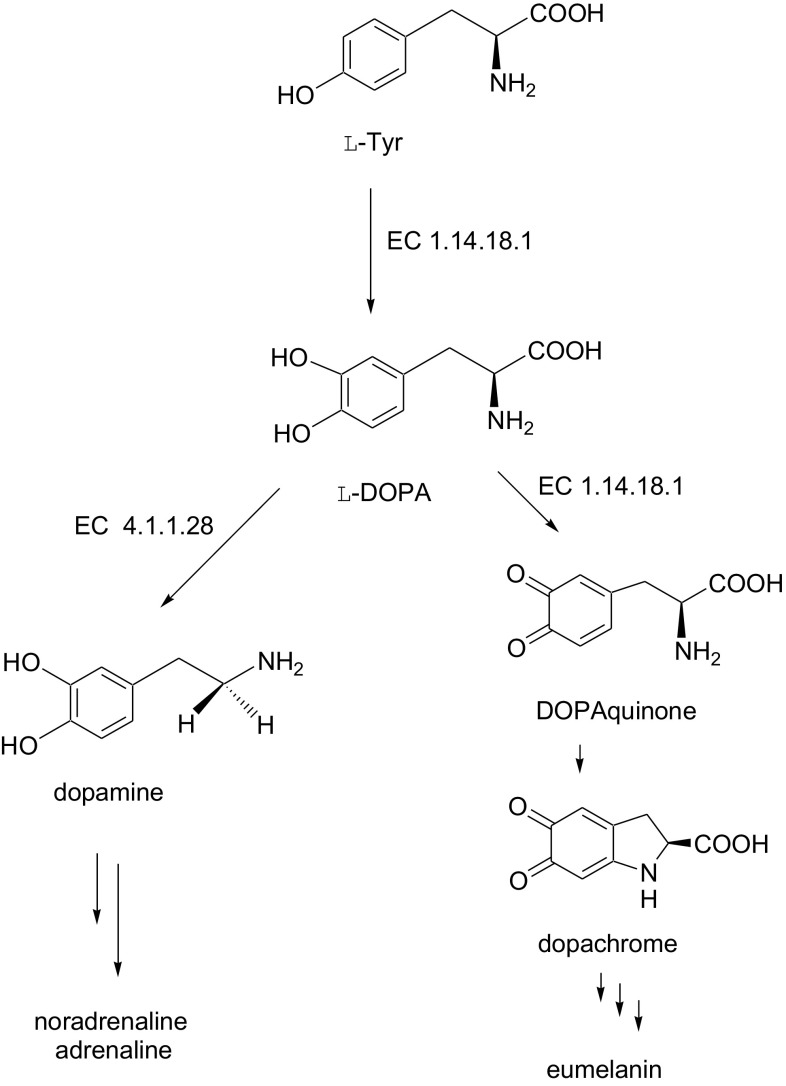
The fragment of l-Tyr metabolic pathway

The enzyme tyrosinase (EC 1.14.18.1) is a copper-containing monooxygenase widely distributed in nature [1, 2]. It is responsible for melanization in animals and enzymatic browning of mushroom, fruit and vegetables in the presence of air. This enzyme catalyses the hydroxylation of monophenol in the *ortho* position (cresolase activity) and oxidation of resulting diphenol to *o*-quinone (catalase activity). Tyrosinase is involved into l-tyrosine (l-Tyr) metabolic pathway where it catalyses hydroxylation of l-Tyr to l-DOPA (3′,4′-dihydroxy-l-phenylalaniane)—the first step of neurotransmitters formation, and oxidation of l-DOPA to dopaquinone (3′,4′-dioxy-l-phenylalanine)—the precursor in melanin formation (Fig. 1). The disturbed metabolism of l-Tyr leads to many diseases including albinism, vitiligo, melanoma or Parkinson’s disease [3–6].

Halogenated derivatives of l-Tyr, labeled with short-lived radioisotopes, have been recently applied in nuclear medicine for diagnosis and treatment of numerous diseases. 2′-[^18^F]Fluoro-l-Tyr and 6′-[^18^F]Fluoro-l-*m*-Tyr are used in positron emission tomography (PET) for measurement of cerebral protein synthesis and to study the dopaminergic system in humans [7, 8]. 2′-[^123^I]Iodo- and 3′-[^125^I]iodo-α-methyl-l-Tyr are developed as tumor imaging agents [9, 10] for single photon emission computed tomography (SPECT). Thus, from medical point of view it is necessary to investigate the metabolism of l-Tyr halogenated derivatives before using such kind of pharmaceuticals in SPECT or PET diagnostics. It is known that iodinated derivatives of l-Tyr are inhibitors of tyrosine hydroxylase (1.14.16.2), the iron-containing monooxygenase which also catalyses hydroxylation of l-Tyr to l-DOPA [11–13]. Therefore, the aim of our studies is to investigate the influence of halogen substitution on enzymatic conversion kinetics of l-Tyr to l-DOPA using kinetic (KIE) and solvent (SIE) isotope effects method. The numerical values of deuterium KIE’s and SIE’s allow to designate the rate determining step and characterize many details of the mechanism of investigated reaction [14, 15].

## Experimental

### Materials

The enzyme mushroom tyrosinase (EC 1.14.18.1, 1715 U/mg) and 3′-iodo-l-tyrosine were from Sigma. Deuterated water (99.9% D) and Amberlite IR-120 (Na^+^) resin were from Aldrich. Silica gel plates (silica gel 60 F_254_) were purchased from Merck. Deuterated 30% KO^2^H/^2^H_2_O and 85% ^2^H_3_PO_4_/^2^H_2_O were obtained from POLATOM, Poland. 3′-Fluoro-l-tyrosine and 3′-chloro-l-tyrosine were purchased from Alfa Aesar.

### Methods

The proton NMR spectra were recorded in D_2_O using tetramethylsilane (TMS) as internal standard on Varian 500 MHz Unity-Plus spectrometer. Chemical shifts are reported in ppm in the following format: chemical shifts, multiplicity (s = singlet, d = doublet, t = triplet, q = quartet), type of hydrogen. In the course of column chromatographic separation the presence of products was checked by TLC using silica gel plates and acetonitrile:water (4:1; v/v) developing solvent (visualization by 0.1% ethanol solution of ninhydrin).

The kinetic assays were performed using Shimadzu-UV-1800 spectrophotometer in plastic UV-cuvette micro (BRAND, Germany).

#### Synthesis of 3′-fluoro-[5′-^2^H]-, **1** and 3′-chloro-[5′-^2^H]-l-Tyr, **2**

20 mg (0.1 mM) of 3′-fluoro-l-Tyr or 20 mg (0.09 mM) of 3′-chloro-l-Tyr was dissolved in 2 mL of 6 M DCl/D_2_O and placed in glass ampoules. The ampoules were connected to a vacuum apparatus, their contents were frozen with liquid nitrogen, outgassed and sealed under vacuum. After thawing, ampoules were placed in a thermostat and kept for 24 h at 120 °C. Next, the residues were lyophilized, dissolved in 2 mL of water and loaded onto a chromatographic column (100 × 10 mm) filled with ion-exchange resin Amberlite IR-120 (H^+^) and deuterium from labile positions was washed out with water. Then products were eluted with 1 M NH_3_ (aq.). The presence of products in each fraction was checked by TLC. The fractions containing products were combined and lyophilized, leaving 11.6 mg (0.057 mmol) of **1** and 14.1 mg (0.065 mmol) of **2** with chemical yields 58 and 70%, respectively.

^1^H NMR (D_2_O, *δ* ppm):

3′-fluoro-l-Tyr: 7.06, dd, $1{\text{H}}_{{2^{\prime}}}$; 6.99, s, $1{\text{H}}_{{5^{\prime}}}$; 6.96, t, $1{\text{H}}_{{6^{\prime}}}$;3.90, q, 1Hα, 3.10, dq, 2H_β_,

3′-fluoro-[5′-^2^H]-l-Tyr, **1**: 7.09, d, $1{\text{H}}_{{2^{\prime}}}$; 6.97, s, $1{\text{H}}_{{6^{\prime}}}$; 4.24, t, 1H_α_; 3.20, dq, 2H_β_,

3′-chloro-l-Tyr: 7.30, d, $1{\text{H}}_{{2^{\prime}}}$; 7.09, dd, $1{\text{H}}_{{6^{\prime}}}$; 6.97, d, $1{\text{H}}_{{5^{\prime}}}$; 3.91, q, 1H_α_; 3.09, dq, 2H_β_,

3′-chloro-[5′-^2^H]-l-Tyr, **2**: 7.25, s, $1{\text{H}}_{{2^{\prime}}}$; 7.06, s, $1{\text{H}}_{{6^{\prime}}}$; 4.23, t, 1H_α_; 3.15, dq, 2H_β_.

#### Determination of H/D KIE for 3′-fluoro-[5′-^2^H]-l-Tyr and 3′-chloro-[5′-^2^H]-l-Tyr

The solutions of 3′-fluoro-[5′-^2^H]-, 3′-chloro-[5′-^2^H]-l-Tyr and enzyme tyrosinase were prepared in 0.1 M phosphate buffer, pH 6.8. Each kinetic experiment consisted of six runs carried out at room temperature in 750 µL plastic cuvette for different concentration of halogenated derivative of l-Tyr (in 0.2–1.2 mM range with 0.2 intervals). The reaction was started by adding to each cuvette 10 µL (17.15 U) solution of enzyme tyrosinase. The progress of hydroxylation was registered spectrophotometrically at *λ*_max_ = 475 nm for 20 min (1 min interval). The increasing absorbance was measured as a result of dopachrome formation. Then, the reaction rates were calculated from the obtained experimental values for different concentration of halogenated l-Tyr and used for the optimization of kinetic parameters (*V*_max_ and *K*_m_) in the Michaelis–Menten equation.

*Determination of SIE for* 3′-*fluoro*- *and* 3′-*chloro*-l-*Tyr* was carried out the similar way as described above. Each kinetic experiment consisted of six runs carried out at room temperature in protonated and deuterated (pD 7.2) media separately. The SIEs were obtained by dividing the values of *V*_max_ and *V*_max_/*K*_m_ for the reaction carried out in water and fully deuterated medium.

#### Determination of tyrosinase inhibition by 3′-iodo-l-Tyr

Each kinetic experiment consisted of six runs carried out at room temperature in 750 µL plastic cuvette for different concentration of l-Tyr (in 0.2–1.2 mM range with 0.2 intervals) in 0.1 M phosphate buffer, pH 6.8. To each cuvette the same amount of 3′-iodo-l-Tyr was added (1, 2 or 3 mM, depending on the experimental series). The reaction was started by adding 10 µL (17.15 U) solution of enzyme tyrosinase. The increasing absorbance was measured spectrophotometrically at *λ*_max_ = 475 nm for 20 min (1 min interval) as a result of dopachrome formation. Then, the kinetic parameters (*V*_max_ and *K*_m_) were calculated and inhibition constants were determined.

## Results and discussion

### Synthesis of 3′-fluoro-[5′-^2^H]-, **1** and 3′-chloro-[5′-^2^H]-l-Tyr, **2**

**Fig. 2:**
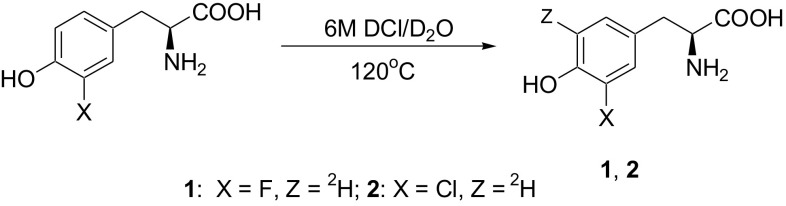
Synthesis of halogenated derivatives of l-Tyr labeled with deuterium in aromatic ring

The deuteration of halogenated derivatives of l-Tyr in aromatic ring was carried out in 6 M DCl/D_2_O at elevated temperature according to procedure described by us earlier [16] (Fig. 2).

The structure and degree of deuterium incorporation was checked by ^1^H NMR spectroscopy and calculated on the basis of signals integration of authentic and deuterated compounds. Disappearance of signals of protons at 5′ position indicates that deuterium enrichment reached almost 100% at the 5′-position of compounds **1** and **2** aromatic rings.

### Determination of isotope effects

The kinetic parameters needed for calculation of deuterium isotope effects i.e., *V*_max_ and *K*_m_ for the reaction of hydroxylation of l-Tyr to l-DOPA (Fig. 1) were determined using non-competitive spectrophotometric method [17]. The values of isotope effects were determined from initial rates (υ) and calculated using computer software Enzfitter 1.05 according to Michaelis [Eq. (1)]

1 $$K_{m} = S\left( {\frac{{V_{\hbox{max} } }}{\upsilon } - 1} \right)$$

where υ is the reaction rate at substrate concentration *S*, *V*_max_ is maximum velocity and *K*_m_ is Michaelis–Menten constant. The experimental errors were calculated using Student’s t-distribution for 95% confidence interval.

**Fig. 3:**
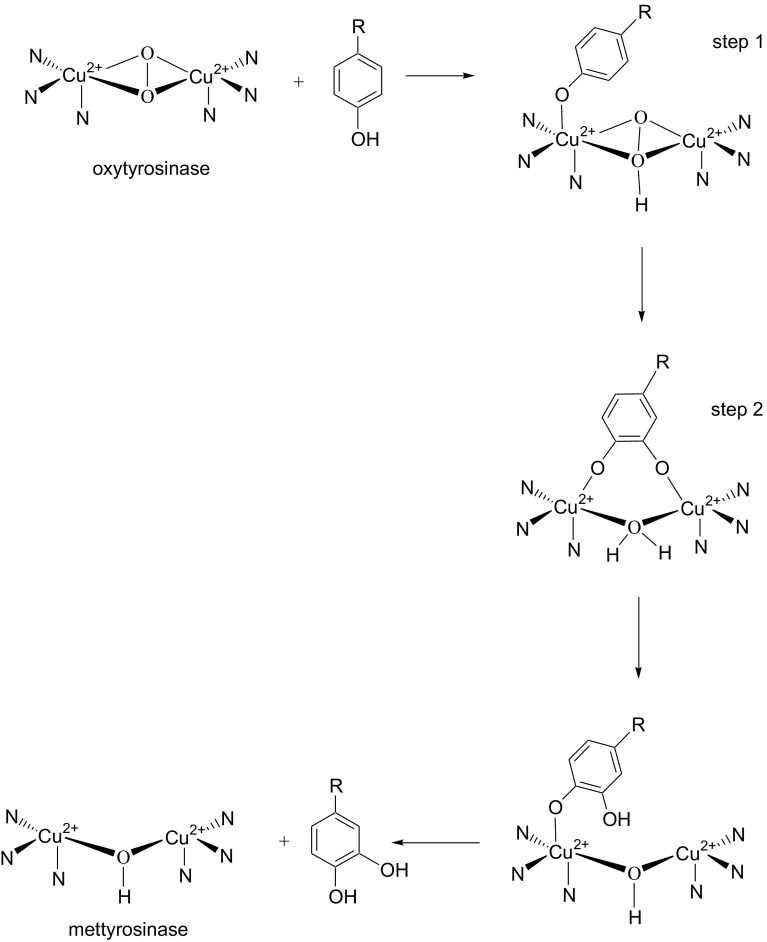
Proposed mechanism for action of tyrosinase [20]

Calculated values of isotope effects are presented in Table 1. Obtained values of KIEs (greater than unity) indicate that the hydrogen atoms in the position C-3 and C-5 of the aromatic ring of halogenated l-Tyr play a role in the conversion of the “enzyme–substrate” complex into “enzyme-product” complex. During the hydroxylation the phenolic substrate coordinates to oxytyrosinase (Fig. 3). In the first step there is the nucleophilic attack of the hydroxyl group of l-Tyr on the copper ion with proton transfer from the OH group of C-4 to the peroxide bound to the copper atom in oxytyrosinase. In the second step electrophilic attack of the peroxide at the C-3 position of l-Tyr is observed [18–20]. The numerical values of KIEs obtained for l-Tyr [20] were greater than or close to 2, which may suggest, that halides donate electron density and hinder the formation of C-O bond, which indicates that this is the rate determining step of investigated reaction (not C-H bond breaking as in case of l-Tyr). That confirms the complexity of this process. SIEs values indicate that solvent have an influence on the proton transfer occurring in this reaction, what is postulated in the literature [21].

**Table 1:** KIE and SIE values for hydroxylation of halogenated derivatives of l-Tyr

Compound	KIE on *V*_max_	KIE on *V*_max_/*K*_m_
3′-Fluoro-l-Tyr/3′-fluoro-[5′-^2^H]-l-Tyr	1.34 ± 0.10	1.65 ± 0.10
3′-Chloro-l-Tyr/3′-chloro-[5′-^2^H]-l-Tyr	1.34 ± 0.07	1.49 ± 0.08

Compound	SIE on *V*_max_	SIE on *V*_max_/*K*_m_
3′-Fluoro-l-Tyr	2.93 ± 0.22	1.84 ± 0.14
3′-Chloro-l-Tyr	1.70 ± 0.09	1.31 ± 0.06

### Determination of tyrosinase inhibition by 3′-iodo-l-Tyr

**Fig. 4:**
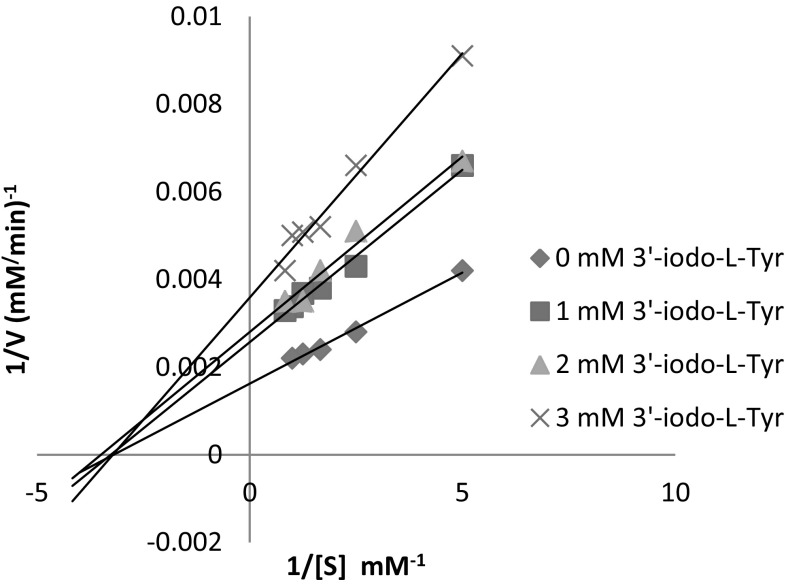
Determination of inhibition type of 3′-iodo-l-Tyr by Lineweaver–Burk plot

We have investigated the inhibitory effect of 3′-iodo-l-Tyr on the hydroxylation of l-Tyr to l-DOPA catalysed by tyrosinase. The type of inhibition was determined from Lineweaver–Burk plots (Fig. 4).

The Lineweaver–Burk plots showed changes in both *V*_max_ and *K*_m_ parameters. The value of *V*_max_ decreases with increasing amount of inhibitor while *K*_m_ parameter increases (Table 2) which indicates that 3′-iodo-l-Tyr induced mixed type of inhibition with competitive inhibition constant *K*_i_ = 5.6 ± 0.9 mM and non-competitive inhibition constant $K^{\prime}_{\text{i}} = 3.3 \pm 0.9\;{\text{mM}}$, calculated from the Eqs. (2) and (3).

2 $$K_{\text{i}} = \frac{{K_{\text{m}} \times [I]}}{{K^{\prime}_{\text{m}} - K_{\text{m}} }}$$

where *K*_*m*_ is the Michaelis constant, $K^{\prime}_{\text{m}}$ is the Michaelis constant with presence of inhibitor, [*I*] is inhibitor concentration

3 $$K^{\prime}_{i} = \frac{{V^{\prime}_{\hbox{max} } \times [I]}}{{V_{\hbox{max} } - V^{\prime}_{\hbox{max} } }}$$

where *V*_max_ is the maximal velocity of the reaction, $V^{\prime}_{\hbox{max} }$ is the maximal velocity of the reaction with presence of inhibitor.

**Table 2:** Values of kinetic parameters for hydroxylation of l-Tyr in presence of 3′-iodo-l-Tyr

Compound	*V*_max_ (mM × min^−1^)	*K*_m_ (mM)
l-Tyr	558 ± 3	0.24 ± 0.02
l-Tyr/1 mM 3′-iodo-l-Tyr	397 ± 33	0.28 ± 0.05
l-Tyr/2 mM 3′-iodo-l-Tyr	359 ± 13	0.32 ± 0.03
l-Tyr/3 mM 3′-iodo-l-Tyr	307 ± 4	0.39 ± 0.01

3′-Iodo-l-Tyr may bond, not only with free enzyme, but also with enzyme–substrate complex. That type of inhibition of tyrosinase is known in literature for some carvacrol derivatives and terephthalic acid [22, 23].

## Conclusions

The aim of this paper was to evaluate the influence of halogen substituent on the kinetics of l-Tyr hydroxylation catalyzed by tyrosinase. The KIE and SIE values obtained in this work are consistent with the mechanism of oxidation of phenolic compounds described earlier [20, 21] and confirms the complex mechanism of action of tyrosinase. Halides donate electron density and hinder the formation of C–O bond during hydroxylation process. Deuterated solvent affect the proton transfer occurring in the first step of investigated reaction. 3′-Iodo-l-Tyr have been found to be an inhibitor of tyrosinase and induced mixed type of inhibition, that is, competitive and non-competitive ones.
